# Illustrations of Medical Botany

**Published:** 1847-12

**Authors:** 


ARTICLE XIV.

*Illustrations of Medical Botany;* consisting of colored figures of the plants affording the most important articles of the Materia Medica, and descriptive letter-press. By Joseph Carson, M. D., professor of Materia Medica in the Philadelphia College of Pharmacy, Member of the American Philosophical Society, of the Academy of Natural Sciences of Philadelphia, Fellow of the College of Physicians, etc. The drawings on stone by J. H. Colen. Volume 1. Philadelphia: Robert P. Smith, 144, Chesnut Street. 1846.

We have received from the publisher a specimen number of the above work, which is now in course of publication. It is to consist of five numbers, large quarto, three of which are now published, and numbers four and five will be shortly. The colored plates are executed with great accuracy, and represent the general appearance with such a degree of correctness, that all acquainted with the plants, would, at first sight, recognize them in the representation. A work of this kind was much needed at the present time, as there is no other of the kind now in print in the United States.

This excellent work not only includes the important medical plants of our own country, but the principal ones of every climate. Each of the plates is accompanied with a botanical history of the plant, and a succinct account of its medicinal properties.

The author, who is well known to the medical profession, is every way qualified to do justice to such an undertaking. It could not have fallen into better hands.

The figures, with the accompanying text, render it most valuable for physicians, and particularly for those whose business it is to teach this branch of medicine. The plates are of such a size, that they can be exhibited to large classes. All who are desirous of being familiar with this department of medicine, would do well to procure the work.

J. McL.

